# Impact of Biochar on Nitrogen-Cycling Functional Genes: A Comparative Study in Mollisol and Alkaline Soils

**DOI:** 10.3390/life14121631

**Published:** 2024-12-09

**Authors:** Junnan Ding, Shaopeng Yu

**Affiliations:** Heilongjiang Province Key Laboratory of Cold Region Wetland Ecology and Environment Research, Harbin University, Harbin 150086, China; wetlands1972@126.com

**Keywords:** nitrogen fixation, nitrification, denitrification, biochar, qPCR

## Abstract

Biochar has gained considerable attention as a sustainable soil amendment due to its potential to enhance soil fertility and mitigate nitrogen (N) losses. This study aimed to investigate the effects of biochar application on the abundance of key N-cycling genes in Mollisol and alkaline soils, focusing on nitrification (AOA, AOB, and *nxrB*) and denitrification (*narG*, *norB*, and *nosZ*) processes. The experiment was conducted using soybean rhizosphere soil. The results demonstrated that biochar significantly altered the microbial community structure by modulating the abundance of these functional genes. Specifically, biochar reduced *narG* and *nosZ* abundance in both soil types, indicating a potential reduction in N_2_O emissions. In contrast, it promoted the abundance of *nxrB*, particularly in alkaline soils, suggesting enhanced nitrite oxidation. The study also revealed strong correlations between N-cycling gene abundances and soil properties such as pH, EC (electrical conductivity. Biochar improved soil pH and nutrient availability, creating favorable conditions for AOB and Nitrospira populations, which play key roles in ammonia and nitrite oxidation. Additionally, the reduction in *norB*/*nosZ* ratios in biochar-treated soils suggests a shift towards more efficient N_2_O reduction. These findings highlight biochar’s dual role in enhancing soil fertility and mitigating greenhouse gas emissions in Mollisol and alkaline soils. The results provide valuable insights into the sustainable management of agricultural soils through biochar application, emphasizing its potential to optimize nitrogen-cycling processes and improve soil health. Further research is needed to explore the long-term impacts of biochar on microbial communities and nitrogen-cycling under field conditions.

## 1. Introduction

Soil microbes play a crucial role in regulating biogeochemical cycles and maintaining ecosystem functions through their involvement in nitrogen cycling [1]. They participate in key ecological processes such as nitrogen fixation, ammonification, nitrification, and denitrification. The composition and function of microbial communities in the nitrogen cycle influence the bioavailability of nitrogen, thereby regulating crop growth characteristics. Moreover, denitrification and ammonia oxidation are the primary contributors to N_2_O emissions, a greenhouse gas with a global warming potential approximately 300 times that of CO_2_ [2]. Therefore, studying the spatial distribution characteristics of nitrogen-cycling microbes is not only a fundamental scientific question regarding microbial evolution and adaptation but also provides a theoretical basis for predicting microbial responses, adaptations, and feedback to changing environmental conditions. Nitrogen is one of the most essential mineral nutrients required for crop growth, but it must undergo transformation in the soil to become absorbable. Soil microbes play a vital role in regulating soil nitrogen-cycling to ensure its availability.

Biochar refers to a highly aromatic, insoluble solid material produced through pyrolysis under completely or partially anaerobic conditions [3]. Typically alkaline, biochar is characterized by its abundant macropores, high specific surface area, diverse surface functional groups, and a large amount of unstable organic matter and nutrients [4,5,6,7,8]. It can be applied to soil as an amendment to alter its physical and chemical properties, such as cation exchange capacity, pH, water retention, bulk density, and carbon and nutrient content, which has attracted significant attention from researchers [9,10,11,12]. Soil nitrogen-cycling is a critical component of element cycling in terrestrial ecosystems, encompassing key processes such as biological nitrogen fixation, ammonification, ammonia volatilization, nitrification, nitrate leaching, and denitrification [13]. Some of these processes, such as nitrogen fixation, nitrification, and denitrification, are regulated by specific microbial functional genes [14]. In recent years, numerous studies have investigated the responses of soil nitrogen-cycling and its functional microbes to biochar application [15]. The results indicate that biochar, by altering soil physical and chemical properties, provides a favorable microenvironment for the growth and reproduction of nitrogen-fixing, nitrifying, and denitrifying bacteria, ultimately influencing soil nitrogen fixation, nitrification, and denitrification processes [16,17,18,19]. An increasing number of reports suggest that biochar can affect soil nitrogen-cycling [20]. Singh et al., through soil column experiments, demonstrated that adding biochar to soil reduces nitrogen leaching [21]. Prommer et al., in field observations of cultivated soils in temperate regions of Europe, found that biochar application promotes soil nitrification [18]. Azeem et al., using isotope tracing technology, revealed that biochar enhances nitrogen fixation rates in root nodules [22]. As research on biochar’s role in regulating soil nitrogen-cycling continues to develop, the microbial mechanisms underlying these effects have become a new focal point. Liu et al. found that adding different proportions of biochar to soil increases the populations of nitrifying and denitrifying bacteria [23]. Chen et al. reported that the addition of biochar significantly enhances the abundance of the ammonia-oxidizing functional gene *aomA* and the dissimilatory nitrate reduction functional gene *nosZ* in farmland soil [24]. Liu et al. observed that biochar application has no significant effect on the abundance of ammonia-oxidizing archaea but consistently increases the community abundance of ammonia-oxidizing bacteria with higher application rates [25]. Abujabhah et al. demonstrated that the relative abundance of nitrogen-fixing bacteria in soil positively correlates with the amount of biochar applied [26]. Additionally, biochar addition significantly suppresses the abundance of denitrification functional genes *narG* and *narH* [27]. Li et al. found that biochar significantly increases the abundance and community size of ammonia-oxidizing bacteria in soil [28]. Recently, significant progress has been made in studying the effects of biochar on nitrogen-cycling functional genes in different soil types. Numerous studies have demonstrated that microbial communities exhibit distinct distribution patterns across various soil types, overturning the traditional hypothesis of random microbial distribution. However, while some research has focused on the distribution characteristics of microbial communities related to individual nitrogen-cycling functional genes, systematic investigations into the distribution patterns and coupling mechanisms of nitrogen-cycling functional genes across different soil types remain limited. Further exploration in this area is essential for understanding the interactions between nitrogen-cycling microbial communities and soil environments.

Northeast China’s Mollisol region is a crucial base for high-quality commercial grain production, contributing approximately 33% of the country’s grain supply and playing a vital role in national food security and ecological stability [29]. The application of biochar has demonstrated significant improvements in the physical and chemical properties of both Mollisol and saline-alkaline soils, enhancing nutrient cycling and soil health [30]. In Mollisols, the biochar increases soil organic matter and total C, N, and P levels, while boosting enzyme activity and microbial biomass, which collectively promote nutrient cycling and availability [31]. It also improves soil structure, enhancing water and nutrient retention, thereby stabilizing the soil ecosystem and increasing crop yields [32]. Notably, the geographic distribution of soil nutrients in Mollisols, particularly organic matter, shows a pattern of higher concentrations in the north and lower levels in the south, making the region an ideal site for studying the biogeographical distribution of soil microbial communities [33,34]. This research has significant implications for improving soil nitrogen use efficiency and reducing negative ecological impacts. In this study, soybean was selected as a model plant because it is the main crop grown in Northeast China, which plays a significant role in the region’s agriculture and economy. The nitrogen-cycling processes in the soybean rhizosphere are representative of the soil nitrogen dynamics in this area. By using soybean rhizosphere soil, the study provides a more accurate evaluation of biochar’s potential to improve soil fertility, optimizing nitrogen-cycling, and reducing greenhouse gas emissions, while also offering valuable insights for the application of biochar in other crops. We hypothesized that: (1) compared to untreated soils, biochar application would enhance the restoration of soil nutrients, including improvements in soil properties and enzyme activities, in both Mollisol and alkaline soils; (2) biochar would induce significant changes in the abundance of nitrogen-cycling functional genes; (3) these variations in nitrogen-cycling gene abundance would be closely associated with changes in soil chemical properties and enzyme activities under different soil types and environmental conditions.

## 2. Materials and Methods

### 2.1. Site Description

Mollisol: the fieldwork took place at the Modern Agricultural Demonstration Park at Heilongjiang Academy of Agricultural Sciences (126°50′ E, 45°50′ N), Harbin, Heilongjiang Province, China. The soil in this region under soil classification is named Mollisol, and the average temperature in the coldest month is −22 °C, while the hottest month has an average temperature of 20 °C, the annual average temperature is ≥10 °C, the annual accumulated temperature is 2000−2800 °C and the frost-free period is 115–130 d, the annual average rainfall is 450−550 mm, of which more than 59% of the rainfall occurs between July and September.

Alkaline soil: the Fanrong village in Zhaodong City (125°34′ E, 46°23′ N), Heilongjiang Province, China. The area of soil is carbonate meadow alkaline soil and carbonate meadow soil. The average annual temperature is 2.4 °C, the annual evaporation is 1662 mm, the average annual rainfall is 396 mm, the maximum temperature is 39.0 °C, the minimum temperature is −37.5 °C, and the annual accumulated temperature is 2500–2700 °C. According to the USDA soil taxonomy system, the soil was predominantly loamy and alkaline experimental, which means a soda saline soil dominated by sodium carbonate bicarbonate [35].

### 2.2. Test Materials

The test biochar is a highly aromatic, carbon-rich material produced from rice husk through pyrolysis and carbonization. It was generated under controlled conditions at a high temperature of specific temperature, e.g., 500 °C for a duration of specific time, e.g., 2 h. Biochar was commercially supplied by Liaoning Golden Future Agriculture Technology Co., Ltd (Shenyang city, China). with the properties of pH 8.69, ≧34% of total nutrient content and N: P_2_O_5_:K_2_O = 8:11:15. The chosen soybean variety was Suinong 35 (*Glycine max* (L.) Merr.). The physiological characteristics of this soybean is an unlimited pod setting habit. The plant is about 90 cm high, with branches, white flowers, long leaves, gray hairs, slightly curved pods, sickle shape, and brown at maturity. The seeds are round, the seed coat is yellow, the navel is light yellow, matte, and the weight of 100 seeds is about 22 g. The protein content was 39.42%, and the fat content was 21.77%. In the adaptive area, the number of days from emergence to maturity is about 120 days, which needs to be ≥10 °C and the active accumulated temperature is about 2450 °C, which was commercially provided by the Soybean Laboratory in the Institute of Tillage and Cultivation, Heilongjiang Academy of Agricultural Sciences.

### 2.3. Experimental Design

In mid-May 2024, the biochar and airdried soil were well-mixed and placed into the pots for soybean pot experiments. Six treatments were set as follows: (1) no biochar was added into the black soil (MB0). (2) 20 g of biochar was added into 1 kg of Mollisol, i.e., 80 g biochar/pot (MB2). (3) 40 g of biochar was added into 1 kg Mollisol, i.e., 160 g biochar/pot (MB4). (4) No biochar was added into the alkaline soil (AB0). (5) 20 g of biochar was added into 1 kg alkaline soil, i.e., 80 g biochar/pot (AB2). (6) 40 g of biochar was added into 1 kg alkaline sol, i.e., 160 g biochar/pot (AB4). Each pot was filled with 3 kg of air-dried and sieved test soil. The corresponding amounts of biochar were thoroughly mixed with the soil, with each treatment replicated three times. Biochar was applied at rates of 0, 20 g·kg^−1^, and 40 g·kg^−1^, which, when converted based on surface area, correspond to 0, 19 t·hm^−2^, and 38 t·hm^−2^, respectively. The soybean seeds were sown on 25 May 2024, with eight seeds per pot. After germination, five uniformly growing seedlings were retained per pot. Each treatment was watered daily with 500 mL using a measuring cup and uniform management practices, including weeding, were applied. No chemical fertilizers or pesticides were used in any treatment.

### 2.4. Soil Sampling

On 12 August 2024, rhizosphere soil was collected using the shaking-off method and placed into sterile plastic bags, which were sealed and quickly transported back to the laboratory in ice boxes. The collected soil samples were then divided into two portions. One portion of the fresh soil was air-dried indoors under natural conditions without exposure to special gasses or dust contamination, then ground, and passed through a 0.25 mm nylon sieve for soil physicochemical property analysis. The other was frozen at −80 °C for DNA extraction, while the other was air-dried at ambient temperature for soil chemical testing.

### 2.5. Soil Chemical Analysis

A pH meter was used to measure the pH of soil samples. The Walkley–Black method was used to determine soil organic matter (SOM) content, then, soil samples were mixed with an excess of potassium dichromate solution and concentrated sulfuric acid to oxidize the organic matter in the soil under acidic conditions. After the oxidation reaction, the remaining dichromate was titrated with a reducing agent (such as sodium oxalate), and the consumption of dichromate was then calculated, and based on the relationship between the dichromate consumption and the standard curve, the SOM content was determined, expressed as a percentage of the soil weight [36]. The methods of concentrated H_2_SO_4_ digestion and Kjeldahl were used to determine the total nitrogen (TN) content of the soil samples [37]. The content of alkali hydrolyzable N (AN) in the soil samples was determined using the alkali hydrolyzable diffusion method [38]. The total content of phosphorus (TP) of the soil samples was determined by HClO_4_ and H_2_SO_4_ digestion molybdenum antimony anti colorimetry. Available phosphorus (AP) in the soil samples was determined by NaHCO_3_ extraction molybdenum antimony anti colorimetry [39]. The total potassium (TK) in the soil samples was determined by digesting soil samples with a mixture of nitric, perchloric, and hydrofluoric acids, followed by flame photometry or atomic absorption spectrophotometry [40]. Available potassium (AK) was extracted using ammonium acetate (1 M, pH 7.0) and quantified using flame photometry or atomic absorption spectrometry [41]. The electrical conductivity (EC) of soil was determined by preparing a saturated soil paste, followed by extracting the solution under a vacuum. The filtrate was then measured using a conductivity meter to quantify the soluble salt concentration [42]. Urease activity is measured by incubating soil with a urea solution and quantifying the ammonium (NH_4_^+^) released using a colorimetric assay [43]. The nitrate reductase activity was determined by incubating soil with potassium nitrate (nitrate of potash), measuring the nitrite produced using diazotization, and coupling with a chromogenic reagent [44]. Cellulase activity was measured by incubating soil with carboxymethyl cellulose as a substrate and quantifying the reducing sugars released, often using the dinitrosalicylic acid (DNS) method [45]. The chitinase activity was determined by incubating soil with colloidal chitin, followed by centrifugation, and measuring the released N-acetylglucosamine via a colorimetric assay [46]. The alkaline phosphatase activity was determined by incubating soil with p-nitrophenyl phosphate (pNPP) as a substrate, followed by measuring the concentration of p-nitrophenol released at 405 nm via spectrophotometry [47]. The catalase activity was determined by incubating soil with hydrogen peroxide (H_2_O_2_) as a substrate, followed by measuring the decrease in absorbance at 240 nm via spectrophotometry [48]. The tyrosinase activity was determined by incubating the sample with L-DOPA as a substrate, followed by measuring the increase in dopachrome concentration at 475 nm via spectrophotometry [49].

### 2.6. Soil DNA Extraction and Quantitative PCR (qPCR)

The Power Soil DNA Isolation Kit was used to extract DNA (MO BIO Laboratories Inc., Carlsbad, CA, USA) was used to extract DNA from 0.25 g of soil in accordance with the manufacturer’s instructions. DNA quality and concentration were determined using a NanoDrop ND2000c spectrophotometer (NanoDrop Technologies, Wilmington, DE, USA) and verified using electrophoresis on a 1% agarose gel. The extracted DNA was subsequently used for quantifying functional genes related to nitrogen cycling in the soil. Quantitative real-time PCR (qPCR) was employed to analyze genes involved in nitrogen fixation (*nifH*), nitrification (*amoA* for bacterial AOB, *amoA* for archaeal AOA, *nxrB*), and denitrification (*narG*, *norB*, *nosZ*), as shown in Table S1. The qPCR reactions were conducted using an ABI7500 Fluorescent Real-Time PCR Detection System (Applied Biosystems, Carlsbad, CA, USA). Specific details about the primers and PCR conditions for each gene can be found in Table S2. All standard curves achieved R^2^ values above 0.99, with slopes ranging from −3.28 to −3.59, and amplification efficiencies between 89.93% and 101.93%.

### 2.7. Statistical Analysis

The Pearson correlation heat maps for soil properties and N-cycling genes were generated using the “pheatmap” package [50]. Additionally, the abundance of different N-cycling genes was ordinated via non-metric multidimensional scaling (NMDS) based on dissimilarity matrices, using the “metaMDS” function from the “vegan” package [51]. A random forest analysis was performed with the “rfPermute” package to evaluate the soil chemical factors driving N-cycling gene abundance [52]. Pearson linear regressions were used to examine correlations between N-cycling gene abundance and significant soil parameters. Using a two-way ANOVA, the impacts of biochar added in Mollisol and alkaline soil on chemical properties and enzyme activity were investigated. Subsequently, the effect of different biochar treatments on soil properties, enzyme activity, N-cycle gene abundance was investigated using a one-way ANOVA. Before the ANOVA, all data were examined using Levene’s test for normality and homogeneity. Duncan’s post hoc test (*p* < 0.05) was used to evaluate group differences. All analyses were performed in R (v.4.2.2) [53]

## 3. Results

### 3.1. Soil Chemical Properties and Soil Enzymes

The presence of biochar addition in Mollisol and alkaline soils can influence soil properties and soil enzymes differently, as shown in Table 1. Table 2 shows that soil enzyme activity is influenced by biochar added in Mollisol and alkaline soil. The results showed that biochar had a significant impact on the pH of both soil types. In Mollisol, the pH fluctuated between 7.38 and 7.45, with minor changes, indicating that biochar had a limited effect on the pH of weakly alkaline soil (Table 1). However, in alkaline soil, biochar application significantly increased pH (*p* < 0.05), especially under the AB2 treatment, which had the highest pH (9.81) (Table 1). This suggests that biochar may have potential for regulating pH balance in strongly alkaline soils. In terms of soil nutrients, biochar significantly increased the SOM, AK, and AN contents in alkaline soil (*p* < 0.05) (Table 1). Notably, in the AB4 treatment, the AK content reached 4668.48 mg/kg, approximately 75% higher than that of the AB0 treatment without biochar (Table 1). Additionally, in Mollisol, the MB2 treatment showed the highest SOM and AN contents, at 46.94 g·kg^−1^ and 95.23 mg·kg^−1^, respectively (Table 1). This indicates that optimized biochar application can significantly enhance soil nutrient content, thereby improving soil fertility. Regarding enzyme activities, the effects of biochar varied across soil types. In Mollisol, the MB4 treatment significantly increased cellulase activity (0.65 µg·10 g^−1^·d^−1^), while in alkaline soil, the AB4 treatment also exhibited significantly higher cellulase activity than other treatments (*p* < 0.05) (Table 1). However, urease and nitrate reductase activities in alkaline soil significantly decreased under the AB4 treatment, to 0.02 mg·g^−1^·d^−1^ and 1.85 mg·g^−1^·d^−1^, respectively (Table 1). This suggests that while biochar generally promotes soil enzyme activities, its effects may be influenced by both soil type and biochar application levels. Results from a two-way ANOVA indicate that biochar significantly improved soil pH (F = 277.328, *p* < 0.001), SOM (F = 19.143, *p* = 0.001), and enzyme activities such as urease (F = 56.812, *p* < 0.001) and alkaline phosphatase (F = 42.79, *p* < 0.001) in both Mollisol and alkaline soils. Significant interaction effects (*p* < 0.01) indicate biochar’s differential impact on nutrient availability (AP, AK) and denitrification regulation via nitrate reductase (Table 2).

The values represented mean ± standard deviations (n = 3). The different letters stand for significant effects (*p* < 0.05). Abbreviations include the following: pH, the soil pH values; SOM, soil organic matter; TN, total nitrogen; TP, total phosphorus; TK, total potassium; AN, available nitrogen; AP, available phosphorus; AK, available potassium; EC, electrical conductivity.

### 3.2. N-Cycling Gene Abundance

**Figure 1:**
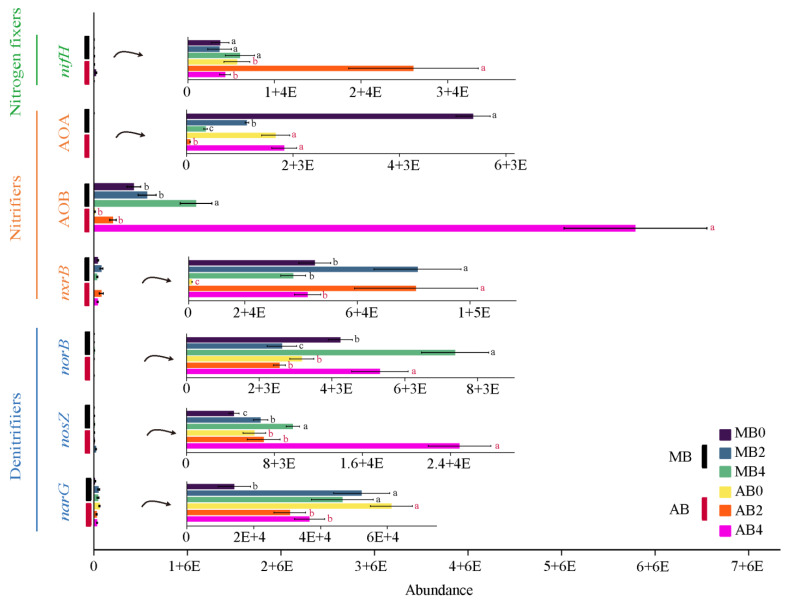
Abundance of nitrogen cycle functional genes in different treatments. Different small letters indicate significant differences between Mollisol×Biochar (MB) and Alkaline soil × Biochar (AB) treatments, respectively (*p* < 0.05).

Biochar treatments had a significant impact on all N-cycling functional genes (Table 3). Figure 1 illustrates the effects of biochar application on nitrogen-cycling microbial abundances in typical Mollisol and alkaline soils in Northeast China, with significant differences between treatments marked. The results show that biochar significantly enhances the abundance of key nitrogen-cycling genes, with notable differences across treatment levels. The nitrogen-fixing gene (*nifH*) exhibits a significant increase under higher biochar levels (MB4 and AB4), with statistical analysis confirming its abundance is significantly greater in these treatments compared to lower levels (*p* < 0.05). Similarly, the abundance of AOB increases significantly with biochar application, particularly at MB4 and AB4, as indicated by significant differences (*p* < 0.05). Conversely, AOA shows a significant decrease in abundance at higher biochar levels at MB4, demonstrating a clear shift in microbial nitrification dynamics. Denitrification-related genes *nosZ* also exhibit significant increases in abundance with biochar, particularly at higher levels, with statistical differences highlighted (*p* < 0.05). This suggests enhanced denitrification potential, which may contribute to reduced nitrous oxide emissions. In contrast, *norB* shows no significant differences across treatments, indicating that its abundance remains stable regardless of biochar application. The nitrite oxidation gene *nxrB* displays its highest abundance in AB2, with significant differences from lower biochar treatments (*p* < 0.05), particularly in alkaline soils, reflecting enhanced nitrite oxidation activity.

### 3.3. N-Cycling Gene Abundance Ratio

**Figure 2:**
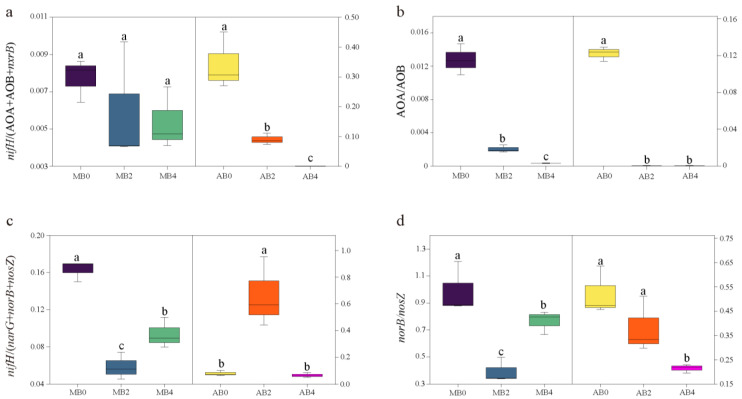
Nitrogen cycles were inferred from the calculation of different nitrogen cycle gene abundance ratios. (**a**–**d**) Represent nitrogen sequestration process, ammonia oxidation process, nitrogen loss process, and N_2_O production process, respectively. Different small letters indicate significant differences between treatments (*p* < 0.05).

Figure 2 illustrates the impact of biochar application on nitrogen-cycling gene abundance ratios in typical Mollisol and alkaline soils, with significant differences highlighted. The *nifH*/(AOA + AOB + nxrB) ratio (Figure 2a), which represents the balance between nitrogen fixation and nitrification, increases significantly with higher biochar levels in both soil types. In Mollisol, MB4 exhibits the highest ratio, which is significantly higher than MB0 and MB2 (*p* < 0.05), indicating that higher biochar levels favor nitrogen fixation. Similarly, in alkaline soils, the AB4 treatment shows a significantly higher ratio compared to AB0 (*p* < 0.05), underscoring biochar’s role in promoting nitrogen fixation across both soils. The AOA/AOB ratio (Figure 2b), reflecting the dominance between archaeal and bacterial ammonia oxidizers, significantly decreases with increasing biochar levels. Both MB4 and AB4 exhibit the lowest ratios, which are significantly different from MB0 and AB0, respectively (*p* < 0.05), suggesting that biochar favors bacterial-driven ammonia oxidation over archaeal processes at higher application levels in both soils. Regarding the *nifH*/(*narG* + *norB* + *nosZ*) ratio (Figure 2c), which compares nitrogen fixation to denitrification potential, no significant differences are observed across biochar treatments in either soil type (*p* > 0.05), indicating a relatively stable balance between these two processes regardless of biochar application level. The *norB*/*nosZ* ratio (Figure 2d), representing the efficiency of nitrous oxide reduction relative to nitric oxide reduction, decreases significantly with higher biochar levels in both soils. In Mollisol, MB4 shows a significantly lower ratio than MB0 and MB2 (*p* < 0.05); while in alkaline soils, AB4 demonstrates a similar reduction compared to AB0 and AB2 (*p* < 0.05), suggesting that biochar enhances the efficiency of nitrous oxide reduction.

### 3.4. Association of Soil Properties and Enzyme Activity with Gene Abundance

**Figure 3:**
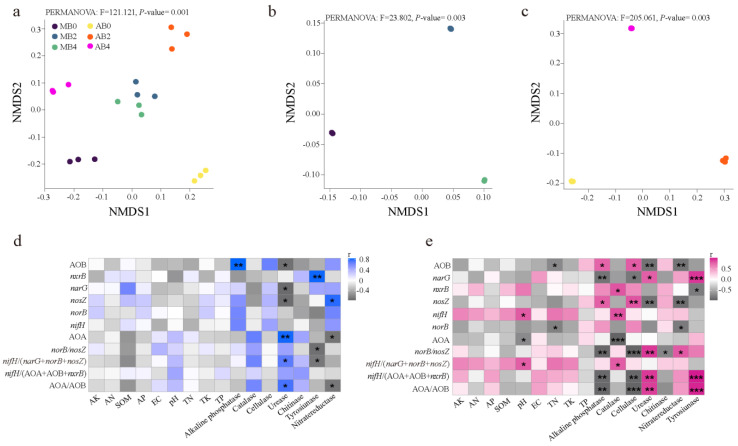
NMDS analysis of treatments and time on functional genes for N-cycling (**a**) and separate analysis of MB (**b**) and AB (**c**). Correlation analysis of soil properties and enzyme activities with nitrogen cycle gene abundance at MB treatment (**d**) and AB treatment (**e**). * *p* < 0.05, ** *p* < 0.01, *** *p* < 0.001.

The effects of biochar application on nitrogen-cycling gene abundances and their correlations with soil properties and enzyme activities are evident in both Mollisol and alkaline soils (Figure 3). The NMDS analysis (Figure 3a) demonstrates a clear differentiation in nitrogen-cycling microbial communities among biochar treatments, with PERMANOVA results confirming these distinctions (F = 121.121, *p* = 0.001). This suggests that biochar markedly reshapes the structure of nitrogen-cycling microbial communities in both soil types. The correlation analysis (Figure 3d) highlights significant relationships between soil properties, enzyme activities, and gene abundances. Specifically, *nosZ* abundance, a key denitrification gene, shows a significant positive correlation with soil pH (*p* < 0.05), suggesting that biochar’s alkalinity-enhancing effect may promote denitrification. Conversely, urease activity correlates negatively with *nosZ* abundance (*p* < 0.05), indicating potential competition between nitrogen mineralization and denitrification pathways. In contrast, AOB abundance negatively correlates with soil EC (*p* < 0.05), indicating sensitivity to salinity under biochar treatments. The *nifH*/(AOA + AOB + *nxrB*) ratio, reflecting the balance between nitrogen fixation and nitrification, shows a significant negative correlation with cellulase activity (*p* < 0.05), suggesting reduced nitrogen fixation in soils with high organic matter decomposition. Meanwhile, the *nifH*/(*narG* + *norB* + *nosZ*) ratio correlates positively with SOM and TP (*p* < 0.05), indicating that biochar may enhance nitrogen fixation potential in soils with higher organic matter and phosphorus content. Further analysis (Figure 3e) shows that *nxrB*, a key nitrification gene, has a significant positive correlation with TP (*p* < 0.05), suggesting that biochar-induced phosphorus availability enhances nitrite oxidation. The *norB*/*nosZ* ratio, which reflects the balance between nitric oxide and nitrous oxide reduction, is positively correlated with EC (*p* < 0.05), indicating that biochar modulates denitrification pathways through its effects on soil conductivity and enzyme activity. These findings demonstrate that biochar significantly influences nitrogen-cycling microbial communities and their interactions with soil properties, optimizing nitrogen-cycling processes in both Mollisol and alkaline soils.

### 3.5. Random Forest Analysis

**Figure 4:**
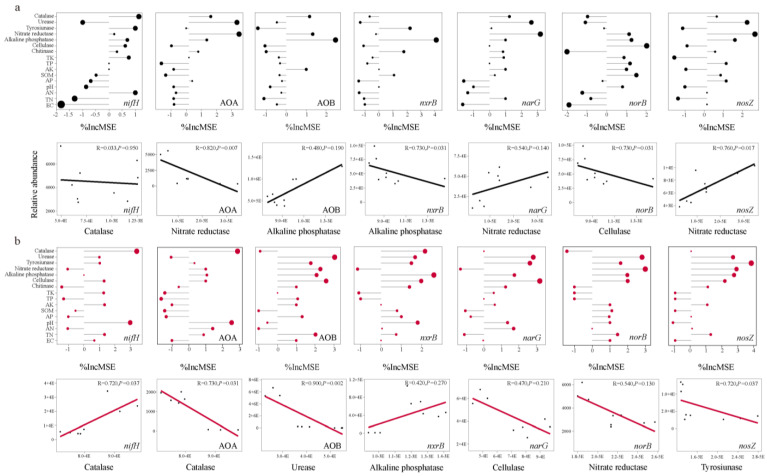
Random forest analysis and Linear regression analysis of soil properties and soil enzyme activity on functional genes abundance of N-cycling at MB treatment (**a**) and AB treatment (**b**). Abbreviations: %IncMSE, the increase in mean square error.

Figure 4 presents the random forest model analysis, which predicts the effects of biochar application on nitrogen-cycling gene abundances in Mollisol and alkaline soils. The model ranks the key predictors for each gene based on their %IncMSE values. The abundances of AOA and *nosZ* are mainly influenced by cellulase activity (Figure 4a), highlighting the crucial role of organic matter decomposition in supporting ammonia oxidation and complete denitrification. The enhancement of cellulase activity suggests that biochar fosters a synergistic relationship between carbon and nitrogen cycling, particularly in reducing greenhouse gas emissions. For AOB and *narG*, soil pH is the most significant predictor (Figure 4a), demonstrating that biochar improves bacterial nitrification and nitrate reduction by creating a more favorable pH environment. This effect is especially pronounced in alkaline soils, emphasizing biochar’s critical role in pH adjustment for optimizing microbial functions. Additionally, the abundance of *nxrB* is primarily driven by nitrate reductase (Figure 4a), suggesting a close linkage between nitrate reduction and nitrite oxidation in the nitrogen cycle. Biochar enhances nitrate reductase activity, thereby increasing the efficiency of nitrite conversion to nitrate and optimizing nitrogen-cycling continuity. Figure 4b shows that under AB treatments, biochar application significantly influences nitrogen-cycling gene abundances, similar to MB treatment, but with a stronger correlation. A key difference in AB treatment is the stronger influence of soil pH on AOB and *narG* gene abundances, suggesting that biochar improves pH conditions, particularly in alkaline soils, to optimize nitrification and nitrate reduction. Finally, *nxrB* abundance is again linked to nitrate reductase activity, indicating that biochar promotes efficient nitrite oxidation and enhances nitrogen-cycling continuity in biochar-amended soils.

## 4. Discussion

### 4.1. Biochar Application Modulates Nitrogen-Cycling Gene Dynamics in Mollisol and Alkaline Soils

Nitrogen is a fundamental nutrient for plant growth, and its deficiency can severely limit agricultural productivity [54]. Biological nitrogen fixation, which depends on nitrogen-fixing microorganisms expressing the *nifH* gene, plays a crucial role in converting atmospheric nitrogen into bioavailable forms that plants can utilize [55]. This process is especially important in sustainable agriculture, where reducing chemical fertilizer dependency is a priority [56]. Our study demonstrated that biochar application significantly altered the abundance of the *nifH* gene in both Mollisol and alkaline soils. These systems exhibited higher *nifH*/(AOA + AOB + *nxrB*) ratios compared to monoculture (Figure 2a), indicating enhanced nitrogen-fixation efficiency. This aligns with previous research, which has shown that biochar improves nitrogen fixation by increasing organic carbon availability and promoting microbial activity [57,58].

In addition to enzymatic processes, soil pH was identified as another critical factor influenced by biochar. Our study revealed that pH significantly affected the abundances of AOB and *narG*, which are crucial for nitrification and nitrate reduction processes, respectively (Figure 4a). Biochar’s liming effect is well-documented, particularly in alkaline soils, where pH modifications can improve microbial habitat conditions and enhance nitrogen-cycling efficiency [59]. Furthermore, a positive correlation was observed between soil pH and *nosZ* abundance (Figure 3d), underscoring biochar’s potential to promote complete denitrification and reduce N_2_O emissions. This finding is consistent with studies by Li et al. [59], who demonstrated that biochar mitigates N_2_O emissions while enhancing nitrogen fixation through improved microbial activity.

The results also indicate that biochar has a broader impact on nitrogen cycling by influencing the relative abundances of multiple functional genes. The enhanced abundance of *nosZ* in biochar-treated soils suggests a shift toward more efficient denitrification pathways, reducing N_2_O accumulation—a potent greenhouse gas. The positive effects of biochar on complete denitrification not only reduce nitrogen losses but also contribute to climate change mitigation by lowering agricultural greenhouse gas emissions. The role of biochar in enhancing nitrogen fixation and cycling was further supported by its impact on microbial community structure. Previous studies have reported that biochar amendments create a more diverse and stable microbial community by providing a rich carbon source and improving soil physical properties [60]. The enhanced nitrogen-fixation efficiency observed in these systems highlights the synergistic effects of biochar and crop diversification [61]. Interestingly, the impact of biochar on nitrogen-cycling processes extends beyond individual genes to the interactions between different microbial pathways. The interplay between nitrification and denitrification processes, mediated by genes such as AOA, AOB, *nxrB*, and *nosZ*, reflects a dynamic balance influenced by soil conditions and biochar amendments. By optimizing this balance, biochar promotes efficient nitrogen use and reduces nitrogen losses through leaching and gaseous emissions. These findings align with broader efforts to enhance soil health and nutrient cycling in agricultural systems [62,63].

Our study demonstrates that biochar significantly influences nitrogen-cycling microbial communities by enhancing enzymatic activities and modifying soil chemical properties. These changes lead to improved nitrogen fixation and cycling efficiencies, particularly in Mollisol and alkaline soils. By reducing N_2_O emissions and supporting sustainable nitrogen management, biochar represents a valuable tool in addressing both agricultural productivity and environmental challenges. These findings provide robust scientific support for integrating biochar into sustainable soil management practices, helping to optimize nitrogen use efficiency and mitigate the environmental impacts of agriculture.

### 4.2. Biochar Application Alters Ecological Niches and Affects Nitrifying Bacteria Abundance in Different Soil Types

The application of biochar significantly alters the ecological niches of nitrifying bacteria, thereby influencing their abundance, activity, and community structure in two distinct soil types common to Northeast China: Mollisol and alkaline soils. These soils, characterized by their unique physical and chemical properties, present varying ecological challenges for microbial communities [64]. Nitrifying bacteria, particularly AOA and AOB, play a pivotal role in soil nitrogen-cycling by converting ammonia to nitrite, a critical step in nitrification [65]. The distinct ecological preferences and functional adaptations of these two microbial groups lead to varied responses under biochar amendments. Our study revealed that biochar application significantly increased AOB abundance in both Mollisol and alkaline soils (Figure 1). This indicates that biochar creates a favorable environment for AOB by enhancing nutrient availability and buffering soil pH. AOB are known to prefer neutral to slightly alkaline conditions, which biochar effectively establishes through its liming effect. This is particularly important in alkaline soils, where natural pH levels can inhibit microbial activity without proper management [66,67].

The increased AOB abundance observed in Mollisol reflects biochar’s role in nutrient retention and organic matter stabilization. In these nutrient-rich soils, AOB thrive under biochar amendments that improve soil structure and nutrient cycling. These results align with previous studies showing that biochar provides a stable carbon matrix and improves oxygen diffusion, promoting bacterial growth and activity [68]. In contrast to AOB, AOA abundance was more pronounced in monoculture systems for both soil types (Figure 1). AOA are known for their ability to adapt to a wider range of soil conditions, including those with lower pH and nutrient availability, making them more competitive in the less diverse monoculture systems of Mollisol and alkaline soils [69]. This adaptability is crucial in acidic patches of Mollisol and nutrient-depleted areas of alkaline soils.

The observed negative correlation between AOA abundance and cellulase activity (Figure 3d) further suggests that AOA thrive in environments with slower organic matter decomposition and lower carbon turnover. This aligns with findings that AOA are more active in carbon-limited conditions, where competition from AOB is less intense [70]. The distinct ecological niches of AOA and AOB are further reflected in the AOA/AOB ratio. In biochar-treated systems, this ratio was significantly lower in both soil types (Figure 2b), indicating a shift toward AOB dominance. This microbial shift improves nitrification efficiency, as AOB are generally more effective in ammonia oxidation under nutrient-rich conditions. In Mollisol, this shift may enhance nitrogen-cycling in soils already rich in organic matter, while in alkaline soils, it helps balance nitrogen dynamics in saline environments [71,72].

The study also highlighted the role of biochar in enhancing the abundance of *nxrB*, a gene encoding nitrite oxidoreductase, which is critical for nitrite oxidation. The application of biochar has been extensively studied for its ability to regulate soil physicochemical properties and influence microbial community dynamics, which directly and indirectly affects soil biogeochemical processes. The results showed that the abundance of the *nxrB* gene in black soil followed the trend MB2 > MB0 > MB4, while in alkaline soil it followed the trend AB2 > AB4 > AB0 (Figure 1). The impact of biochar application rates on nxrB gene abundance can be attributed to changes in soil properties, microbial habitat, and nutrient supply. The increased abundance of *nxrB* genes observed in MB2 and AB2 treatments can be attributed to the optimal application of biochar, which enhances the microenvironment favorable for the growth of nitrifying bacteria containing the nxrB gene. Moderate biochar application may improve soil aeration, water retention, and the supply of essential nutrients (e.g., carbon sources and mineral nitrogen) required by nitrifying bacteria [73]. Black soil and alkaline soil in Northeast China have different physicochemical properties, and moderate biochar application can improve the pH buffering capacity of alkaline soil and improve the structure of black soil, thereby providing better conditions for microbial growth. Biochar can also increase soil pH, which may be particularly beneficial for nitrifying bacteria in acidic soils, thereby supporting the growth and activity of *nxrB*-containing bacteria [74]. Additionally, biochar provides a physical habitat for microorganisms, which can serve as a niche for the growth and colonization of nitrite-oxidizing bacteria, thereby increasing the abundance of the *nxrB* gene [75]. However, a decrease in *nxrB* gene abundance was observed at higher application rates (MB4 and AB4). Excessive biochar application may create unfavorable conditions for microbial communities, including nitrifying bacteria. High doses of biochar may lead to an increase in soil alkalinity, exceeding the tolerance range of many nitrifying microorganisms. Additionally, a large amount of biochar may increase soil pore space, leading to desiccation or water retention imbalance, which may create an environment unfavorable for microbial activity [75]. In the alkaline soil of Northeast China, excessive biochar may further exacerbate the degree of salinization, inhibiting microbial activity. High biochar content may also increase the adsorption of ammonium and nitrite, thereby reducing substrate availability for nitrite-oxidizing bacteria [73]. Furthermore, excessive biochar may lead to an imbalance in the carbon-to-nitrogen ratio, triggering competition among microorganisms or inhibitory effects due to volatile organic compounds present in biochar, thereby suppressing bacterial populations containing the *nxrB* gene [75].

The monoculture systems exhibited higher *nxrB* abundance in certain cases, particularly in alkaline soils, where reduced microbial competition and increased nitrite availability may have supported Nitrospira activity. Moreover, the positive correlation between *nxrB* abundance and TP content (Figure 3d) underscores the nutrient-driven dynamics of nitrite oxidation. Biochar’s ability to increase phosphorus availability in Mollisol and mitigate phosphorus fixation in alkaline soils further enhances Nitrospira proliferation [76]. The enhanced nitrification observed in biochar-treated Mollisol and alkaline soils has significant implications for soil fertility and agricultural productivity in Northeast China. By promoting AOB dominance and increasing *nxrB* activity, biochar improves nitrogen-cycling efficiency, reducing nitrogen losses through leaching and gaseous emissions. This is particularly critical in alkaline soils, which are prone to nitrogen volatilization due to high pH, and in Mollisol, where nitrogen leaching is a common challenge due to high organic matter content.

Furthermore, the ability of biochar to modulate microbial community dynamics underscores its potential as a sustainable soil amendment. By creating more favorable ecological niches for nitrifying bacteria, biochar supports a balanced nitrogen cycle that enhances crop nutrient uptake while mitigating environmental impacts [77]. This dual benefit highlights biochar’s role in advancing sustainable agricultural practices, particularly in regions with diverse soil types and cropping systems. Our findings demonstrate that biochar application significantly reshapes the ecological niches of nitrifying bacteria in Mollisol and alkaline soils. By promoting AOB and Nitrospira activity, biochar optimizes nitrogen-cycling processes, contributing to improved soil health and agricultural productivity. Future research should investigate the long-term impacts of biochar on microbial community dynamics and explore its integration into comprehensive soil management strategies for sustainable agriculture.

### 4.3. Biochar Application Modulates narG and nosZ Abundance to Influence Nitrogen Losses in Mollisol and Alkaline Soils

This study highlights the significant influence of biochar on the abundance of two key denitrification genes, *narG* and *nosZ,* in Mollisol and alkaline soils. These genes are critical in regulating nitrogen losses through denitrification, a process that reduces nitrate to gaseous nitrogen compounds, including nitrous oxide (N_2_O), a potent greenhouse gas. The findings reveal that biochar application can modulate the activity of microbial communities involved in denitrification, thereby improving nitrogen retention and reducing environmental nitrogen losses, but the long-term effects of biochar on soil fertility, microbial diversity, and nutrient cycling need further investigation. Moreover, the economic feasibility of large-scale biochar application remains a critical factor in determining its practical use in agriculture [78]. The abundance of *narG*, which encodes nitrate reductase responsible for converting nitrate to nitrite, was significantly lower in biochar-treated soils compared to untreated controls (Figure 1). This reduction is particularly noteworthy in Mollisol, where the higher organic matter content and microbial diversity create a more competitive environment for denitrifying bacteria [79]. The decrease in *narG* abundance in biochar-amended soils is consistent with findings by Gou et al., who reported a similar reduction in nitrate reductase activity under improved soil aeration conditions [80]. Biochar’s porous structure enhances soil aeration, thereby reducing the anaerobic niches necessary for nitrate reduction. In alkaline soils, biochar application not only improved soil structure but also mitigated the negative impacts of high salinity, which often hampers microbial activity. This was reflected in a moderate reduction in *narG* abundance. The correlation between *narG* abundance and EC (Figure 3e) suggests that biochar’s ability to lower EC in saline soils plays a crucial role in modulating denitrification dynamics [81].

The gene *nosZ*, encoding nitrous oxide reductase, catalyzes the final step of denitrification, converting N_2_O to nitrogen gas (N_2_) [82]. Its abundance was significantly reduced in biochar-treated soils, particularly in Mollisol, where biochar raised soil pH levels (Figure 3d and Table 1). Previous research indicates that *nosZ* abundance is sensitive to pH changes, with lower abundances observed in more alkaline environments [83]. This reduction may initially suggest an increased potential for N_2_O accumulation; however, biochar’s impact on soil moisture and microbial habitat can mitigate such risks. Notably, the *norB*/*nosZ* ratio was significantly lower in biochar-treated soils (Figure 2d), indicating an enhanced capacity for N_2_O reduction relative to its production. This finding aligns with studies demonstrating that biochar improves the efficiency of microbial N_2_O sinks by supporting organisms expressing *nosZ* [84,85,86]. Castellano et al. observed a similar enhancement in denitrifying bacteria populations in soils with improved water retention and nutrient availability, conditions promoted by biochar application [87].

The observed changes in *narG* and *nosZ* abundances also reflect biochar’s influence on soil moisture dynamics. By improving soil water retention, biochar creates conditions that can limit denitrification in surface soils while promoting it in deeper layers [88]. This is particularly evident in alkaline soils, where biochar-treated soils showed reduced moisture levels, enhancing microbial activity and supporting a diverse denitrifying community. The modulation of *narG* and *nosZ* abundance by biochar has significant implications for nitrogen-cycling in agricultural soils. By reducing the abundance of *narG*, biochar limits nitrate reduction, which in turn reduces the substrate availability for subsequent denitrification steps. Similarly, the regulation of *nosZ* abundance helps to mitigate N_2_O emissions, particularly in Mollisol, where biochar’s impact on microbial community dynamics is more pronounced. These findings underscore biochar’s potential as a sustainable soil amendment that enhances nitrogen use efficiency while minimizing environmental impacts [88]. The reduction in nitrogen losses and the mitigation of N_2_O emissions contribute to improved soil fertility and support sustainable agricultural practices in regions characterized by Mollisol and alkaline soils.

## 5. Conclusions

This study, conducted using a pot experiment, investigated the impact of biochar on nitrogen-cycling processes in Mollisol and alkaline soils. The results demonstrate that biochar significantly regulates the abundance of key denitrification genes and alters microbial community dynamics, thereby optimizing nitrogen-cycling processes. Biochar effectively reduces the abundance of *narG* and *nosZ* in surface soils, indicating its potential to mitigate nitrogen losses and control N_2_O emissions. However, in alkaline soils, biochar’s effect on soil salinity and pH creates a more favorable environment for denitrifying bacteria, resulting in enhanced nitrogen-cycling efficiency. The abundance of *narG* was strongly influenced by EC, highlighting the role of biochar in improving soil structure and mitigating salinity stress. Similarly, *nosZ* abundance was modulated by soil pH, emphasizing biochar’s impact on nitrogen reduction processes and its potential to regulate N_2_O emissions. These findings demonstrate biochar’s capacity to enhance nitrogen retention and reduce greenhouse gas emissions by improving soil chemical properties and microbial functionality. The study also revealed that biochar supports a more diverse and competitive microbial environment in both soil types. In Mollisol, biochar enhances organic matter stabilization and improves nutrient availability, while in alkaline soils, it alleviates salinity stress and promotes microbial resilience. These changes lead to a more balanced nitrogen cycle, reducing nitrogen losses and optimizing soil fertility. Overall, biochar proves to be a sustainable soil amendment with significant potential for improving nitrogen use efficiency and reducing environmental impacts in agricultural systems. Future research should focus on long-term field trials to assess the continued impact of biochar on microbial community dynamics and nitrogen-cycling processes over time. Moreover, studies should explore biochar’s integration into broader soil management practices, considering regional variations in soil types, climate conditions, and agricultural practices. Such research will provide critical insights into the potential for biochar to serve as a sustainable solution for improving nitrogen use efficiency and minimizing environmental impacts in agriculture.

## Figures and Tables

**Table 1:** The soil chemical properties and soil enzymes in two soils with different biochar application.

	Mollisol × Biochar			Alkaline Soil × Biochar		
	MB0	MB2	MB4	AB0	AB2	AB4
pH	7.45 ± 0.00 a	7.38 ± 0.11 a	7.41 ± 0.05 b	9.16 ± 0.32 b	9.81 ± 0.19 a	9.19 ± 0.31 b
SOM (g·kg^−1^)	39.02 ± 0.66 b	46.94 ± 3.72 ab	38.53 ± 4.51 a	15.81 ± 4.38 b	21.43 ± 4.04 a	25.90 ± 3.72 a
TN (g·kg^−1^)	0.51 ± 0.13 a	0.55 ± 0.07 a	0.44 ± 0.16 a	0.45 ± 0.08 a	0.49 ± 0.01 a	0.27 ± 0.04 b
TP (g·kg^−1^)	4.23 ± 0.98 b	5.13 ± 0.90 a	4.60 ± 0.13 b	4.83 ± 0.98 a	3.70 ± 0.10 b	1.60 ± 0.13 c
TK (g·kg^−1^)	3.21 ± 0.51 b	3.62 ± 0.98 ab	4.34 ± 0.55 a	3.72 ± 0.16 b	4.67 ± 068 a	4.31 ± 0.43 a
AN (mg·kg^−1^)	88.32 ± 10.53 b	95.23 ± 12.11 b	111.95 ± 9.19 a	57.14 ± 7.89 c	67.39 ± 6.67 b	78.07 ± 4.09 a
AP (mg·kg^−1^)	61.95 ± 11.53 a	52.31 ± 14.57 a	69.24 ± 29.00 a	49.16 ± 2.07 b	58.57 ± 0.93 a	39.31 ± 4.35 c
AK (mg·kg^−1^)	3210.73 ± 516.19 b	3619.71 ± 983.01 b	4345.11 ± 554.75 b	2667.54 ± 249.07 c	3718.73 ± 364.56 b	4668.48 ± 266.46 a
EC (mS·cm^−1^)	11.22 ± 5.28 a	10.55 ± 1.81 b	9.11 ± 4.03 c	11.33 ± 5.62 a	7.55 ± 3.71 ab	5.02 ± 1.01 b
Urease (mg·g^−1^·d^−1^)	0.05 ± 0.00 a	0.05 ± 0.00 a	0.04 ± 0.00 b	0.05 ± 0.00 a	0.03 ± 0.00 b	0.02 ± 0.00 c
Nitrate reductase (mg·g^−1^·d^−1^)	5.25 ± 0.02 b	1.37 ± 0.03 b	2.37 ± 0.01 a	2.32 ± 0.01 b	2.24 ± 0.01 c	1.85 ± 0.04 a
Cellulase (µg·10 g^−1^·d^−1^)	0.27 ± 0.05 c	0.57 ± 0.02 b	0.65 ± 0.02 a	0.16 ± 0.01 c	0.21 ± 0.01 b	0.41 ± 0.02 a
Chitinase (mU·g^−1^·d^−1^)	0.27 ± 0.06	0.26 ± 0.01	0.21 ± 0.02	0.19 ± 0.01	0.22 ± 0.02	0.22 ± 0.01
Alkaline phosphatase (mg·g^−1^·h^−1^)	0.89 ± 0.03 b	0.80 ± 0.02 c	1.16 ± 0.00 a	0.97 ± 0.02 c	1.21 ± 0.03 b	1.35 ± 0.05 a
Catalase (mL·g^−1^·h^−1^)	1.14 ± 0.09 a	0.67 ± 0.01 c	0.78 ± 0.00 b	0.76 ± 0.03 b	0.92 ± 0.04 a	0.75 ± 0.02 b
Tyrosinase (µmol·g^−1^·min^−1^)	2.23 ± 0.33	2.22 ± 0.84	5.13 ± 0.15	2.44 ± 0.27	1.40 ± 0.47	1.29 ± 0.17

Values represented mean ± standard deviations (n = 3). Different letters stand for significant effects (*p* < 0.05). Abbreviations, SOM, soil organic matter; TN, total nitrogen; TP, total phosphorus; TK, total potassium; AN, available nitrogen; AP, available phosphorus; AK, available potassium; EC, electrical Conductivity.

**Table 2:** Two-way ANOVAs for the impact of Mollisol × Biochar (MB), Alkaline soil × Biochar (AB) and their interaction (MB × AB) on soil chemical properties and soil enzymes.

	MB		AB		MB × AB	
	*F*	*p*	*F*	*p*	*F*	*p*
pH	277.328	<0.001	2.775	0.102	3.604	0.059
SOM	19.143	0.001	2.543	0.121	0.762	<0.001
TN	1.623	0.227	2.515	0.122	0.247	<0.001
TP	0.396	0.054	0.581	0.057	0.658	0.053
TK	0.019	0.891	0.196	0.824	0.694	0.519
AN	4.472	0.056	0.675	0.527	0.124	0.884
AP	1.577	0.233	0.085	<0.001	0.534	<0.001
AK	0.103	<0.001	0.323	<0.001	0.999	0.397
EC	1.036	0.329	1.134	0.354	0.303	0.744
Urease	56.812	<0.001	49.269	<0.001	8.958	0.004
Nitrate reductase	7.422	0.018	2.394	0.133	6.753	0.011
Cellulase	2.574	0.135	6.904	0.01	2.143	0.16
Chitinase	4.505	0.055	0.63	0.55	2.192	0.154
Alkaline phosphatase	42.79	<0.001	15.01	<0.001	26.8	<0.001
Catalase	0.387	0.545	1.931	0.188	4.885	0.028
Tyrosinase	18.492	<0.001	13.59	<0.001	3.398	0.068

**Table 3:** Two-way ANOVAs for the impact of Mollisol×Biochar (MB), Alkaline soil×Biochar (AB) and their interaction (MB × AB) on N-cycling gene abundance.

	MB		AB		MB × AB	
	*F*	*p*	*F*	*p*	*F*	*p*
*nifH*	23.82	<0.001	18.405	<0.001	23.57	0.01
AOA	142.81	<0.001	386.74	<0.001	264.48	<0.001
AOB	73.55	<0.001	188.93	<0.001	123.95	<0.001
*nxrB*	5.77	0.033	40.63	<0.001	8.07	0.006
*narG*	2.82	0.11	0.69	<0.001	46.09	<0.001
*norB*	16.35	0.002	72.06	<0.001	4.82	0.029
*nosZ*	72.51	<0.001	123.09	<0.001	48.61	<0.001

## Data Availability

The datasets generated during and/or analyzed during the current study are available from the corresponding author upon reasonable request.

## Supplementary Materials

The following supporting information can be downloaded at: https://www.mdpi.com/article/10.3390/life14121631/s1, Table S1: Summary of genes investigated, the enzymes they encode, and their function in the nitrogen cycle; Table S2: PCR primers used for quantitative PCR and reaction conditions. References [89,90,91,92,93,94,95] are cited in the Supplementary Materials.
