# Supplementary material for: Impact of Biochar on Nitrogen-Cycling Functional Genes: A Comparative Study in Mollisol and Alkaline Soils
Source: Life (Basel). 2024 Dec 9;14(12):1631. doi: 10.3390/life14121631 (PMC11677638; doi:10.3390/life14121631)
Supplement: Supplementary file 1 [file life-14-01631-s001.zip › life-3347735-supplementary.pdf]

Table S1. Summary of genes investigated, the enzymes they encode, and their function in the nitrogen cycle.

Table S2. PCR primers used for quantitative PCR and reaction conditions.

Table S1

	Enzyme	Process (es)	Details
nifH	Nitrogenase	Nitrogen fixation	Reduction of N_2 to NH_4^+
amoA	Ammonia monooxygenase	Aerobic ammonia oxidation	Oxidation of NH_4^+ to NH_2OH
nxrB	Nitrite oxidoreductase enzyme	Nitrification	Oxidation of NO_2^- to NO_3^-
narG	NO_3^- reductase	Denitrification	Reduction of NO_3^- to NO_2^-
norB	NO reductase	Denitrification	Reduction of NO to N_2O
nosZ	N_2O reductase	Denitrification	Reduction of N_2O to N_2

Table S2

Target gene	Primers	Primer Sequence	Reaction	Cycling conditions	Reference
nifH	nifH-F<br>nifH-R	5′-AAAGGYGGWATCGGYAARTCCACCAC-3′<br>5′-TTGTTSGCSGCRTACATSGCCATCAT-3′	16.5 μL of 2 X ChamQ SYBR® Color qPCR Master MixTM, 2 μL of DNA template, and 0.8 μL of each primer.	95°C-5 min.; 40x 95°C-5s, 55°C-30s, 72°C-40s	[89]
AOA *amo*A	Arch-amoAF<br>Arch-amoAR	5′-STAATGGTCTGGCTTAGACG-3′<br>5′-GCGGCCATCCATCTGTATGT-3′	16.5 μL of 2 X ChamQ SYBR® Color qPCR Master MixTM, 2 μL of DNA template, and 0.8 μL of each primer.	95°C-5 min.; 40x 95°C-5s, 58°C-30s, 72°C-40s	[90]
AOB *amoA*	AmoA-1F<br>AmoA-2R	5′-GGGGTTTCTACTGGTGGT-3′<br>5′-CCCCTCKGSAAAGCCTTCTTC-3′	16.5 μL of 2 X ChamQ SYBR® Color qPCR Master MixTM, 2 μL of DNA template, and 0.8 μL of each primer.	95°C-5 min.; 40x 95°C-5s, 58°C-30s, 72°C-40s	[91]
norB	norB-F<br>norB-R	5′-AAATGGCTTTACGTCATCGTCG-3′<br>5′-TCTGCGTGCCGTGGGTGT-3′	16.5 μL of 2 X ChamQ SYBR® Color qPCR Master MixTM, 2 μL of DNA template, and 0.8 μL of each primer.	95°C-5 min.; 40x 95°C-5s, 60°C-30s, 72°C-40s	[92]
nosZ	nosZ-F<br>nosZ-R	5′-CGYTGTTCMTCGACAGCCAG-3′<br>5′-CATGTGCAGNGCRTGGCAGAA-3′	16.5 μL of 2 X ChamQ SYBR® Color qPCR Master MixTM, 2 μL of DNA template, and 0.8 μL of each primer.	95°C-5 min.; 40x 95°C-5s, 60°C-30s, 72°C-40s	[93]
narG	narG-f<br>narG-r	5'- TCGCCSATYCCGGCSATGTC-3'<br>5'- GAGTTGTACCAGTCRGCSGAYTCSG-3'	16.5 μL of 2 X ChamQ SYBR® Color qPCR Master MixTM, 2 μL of DNA template, and 0.8 μL of each primer.	95°C-5 min.; 40x 95°C-5s, 65°C-30s, 72°C-40s	[94]
nxrB	nxrB-F<br>nxrB-R	5'- TACATGTGGTGGAACA-3'<br>5'- CGGTTCTGGTCRATCA-3'	16.5 μL of 2 X ChamQ SYBR® Color qPCR Master MixTM, 2 μL of DNA template, and 0.8 μL of each primer.	95°C-5 min.; 40x 95°C-5s, 50°C-30s, 72°C-40s	[95]
